# The Effect of Chronic Mild Stress and Imipramine on the Markers of Oxidative Stress and Antioxidant System in Rat Liver

**DOI:** 10.1007/s12640-016-9614-8

**Published:** 2016-03-10

**Authors:** Weronika Duda, Katarzyna Curzytek, Marta Kubera, Małgorzata Iciek, Danuta Kowalczyk-Pachel, Anna Bilska-Wilkosz, Elżbieta Lorenc-Koci, Monika Leśkiewicz, Agnieszka Basta-Kaim, Bogusława Budziszewska, Magdalena Regulska, Joanna Ślusarczyk, Piotr Gruca, Mariusz Papp, Michael Maes, Władysław Lasoń, Lucyna Antkiewicz-Michaluk

**Affiliations:** Department of Experimental Neuroendocrinology, Institute of Pharmacology, Polish Academy of Sciences, 12 Smętna Street, 31-343 Kraków, Poland; Institute of Medical Biochemistry, Jagiellonian University, Medical College, 7 Kopernika Street, 31-034 Kraków, Poland; Department of Neuro- and Psychopharmacology, Institute of Pharmacology, Polish Academy of Sciences, 12 Smętna Street, 31-343 Kraków, Poland; Behavioral Pharmacology Laboratory, Institute of Pharmacology, Polish Academy of Sciences, 12 Smętna Street, 31-343 Kraków, Poland; Department of Psychiatry, Faculty of Medicine, Chulalongkorn University, Bangkok, 10330 Thailand; Department of Neurochemistry, Institute of Pharmacology, Polish Academy of Sciences, 12 Smętna Street, 31-343 Kraków, Poland

**Keywords:** Chronic mild stress, Depression, Imipramine, Liver, Oxidative stress

## Abstract

Liver abnormalities have been reported to occur in up to 20 % of patients on a long-term therapy with the tricyclic antidepressant drug imipramine (IMI). The mechanism involved in this IMI-induced process is unknown but a contribution of oxidative stress is highly likely. Chronic mild stress (CMS) is widely used for modeling depressive-like behavior in rats. In the present study, we examined the effects of CMS and chronic IMI treatment, applied alone or in combination, on the levels of oxidative stress markers, such as reactive oxygen species (ROS), malondialdehyde (MDA), non-protein sulfhydryl groups, and sulfane sulfur as well as on activities of key antioxidant enzymes: catalase (CAT), glutathione peroxidase (GPx), and superoxide dismutase in the rat liver. Administration of IMI for 5 weeks to rats subjected to CMS resulted in a gradual significant reduction of anhedonia measured by sucrose intake, in a majority of animals (CMS IMI-reactive, CMS IMI-R), although about 20 % of rats did not respond to the IMI treatment (CMS IMI non-reactive, CMS IMI-NR). CMS-induced hepatic oxidative stress, estimated by increased ROS and MDA concentrations, was not prevented by the IMI administration, moreover, in CMS IMI-NR animals, the level of the marker of lipid peroxidation, i.e., MDA was increased in comparison to CMS-subjected rats and activity of antioxidant enzymes (GPx and CAT) was decreased compared to IMI-treated rats. The clinical significance of this observation remains to be established.

## Introduction

Clinical reports suggest a close relationship between stressors, particularly those of long duration, and liver diseases, such as hepatic inflammation, that is proposed to be caused by reactive oxygen species (ROS). The liver exhibits one of the highest antioxidant enzyme capacities in the body due to its major metabolic roles including glycogen storage, decomposition of red blood cells, plasma protein synthesis, detoxification, and others (Navarro-Arevalo and Sanchez-Del-Pino 1998). This organ also plays indispensable role in the adaptive response to neuroendocrine stress, when its anabolic activity provides energy-rich compounds, such as glucose and lipids, necessary for adaptation of the body.

Tricyclic/tetracyclic antidepressant drugs are known for their hepatotoxicity but only few cases were reported lately, possibly due to their relatively infrequent current use in the USA and Europe (Sedky et al. 2012; El-Hage et al. 2013). Causality is not well established due to concurrent administration of other drugs and/or underlying liver disease (DeSanty and Amabile 2007). Patients treated with tricyclic drugs are at a greater risk of liver damage than those receiving newer antidepressant agents (Lucena et al. 2003). Imipramine (IMI) is among the antidepressants associated with a greater risk of hepatotoxicity since the drug was introduced to the therapy in 1957 (Moskovitz et al. 1982; Voican et al. 2014). IMI is a dibenzazepine-derived tricyclic antidepressant which acts by serotonin and norepinephrine reuptake inhibition within the synaptic cleft in the central nervous system. IMI is still widely used, more than 1 million prescriptions being filled yearly. IMI is indicated not only for therapy of depression but also is used for childhood enuresis. Liver test abnormalities have been reported to occur occasionally in up to 20 % of patients on a long-term IMI therapy (Moskovitz et al. 1982). For instance, a prolonged cholestatic hepatitis development was described in an IMI-treated person (Horst et al. 1980). The mechanism by which IMI causes liver test abnormalities is not known but probably oxidative stress is involved in this process.

In experimental animals, a chronic mild stress (CMS) paradigm is considered to be a model of depression and is widely used for examining the effects of treatment with antidepressant drugs (Willner 2005). Exposure to CMS produces a markedly diminished interest in rewarding stimuli, evidenced by reduced preference for a palatable sucrose solution over water (Papp et al. 1991; Kubera et al. 1996). This represents a disturbance of the ability to experience pleasure which is suggested to model human anhedonia, a core symptom of major depression episodes according to DSM-IV criteria (Diagnostic and Statistical Manual of Mental Disorders Fourth Edition, American Psychiatric Association 1994; Willner 2005). Repeated antidepressant treatments antagonize stress-induced anhedonia (Papp and Moryl 1994; Kubera et al. 2001) and also behavioral passivity in the forced swimming test (Rogóż et al. 2009), although in our studies some of us established that a part of animals did not respond to IMI treatment in the CMS model of depression (Faron-Górecka et al. 2014). It is unknown why in some rats exposed to CMS IMI does not produce a therapeutic effect. It is similar to clinical situation, when some group of patients does not respond to antidepressive drugs (El-Hage et al. 2013).

Some recent studies strongly suggested that oxidative stress plays a significant role in the stress-induced depressive illness (Khanzode et al. 2003; Sarandol et al. 2007; Lucca et al. 2009a, b; Maes et al. 2011b; Stefanescu and Ciobica 2012). If so, it is reasonable to think that chronic antidepressant treatment paralleled by normalization of depressive-like symptoms would be expected to reverse effects of oxidative stress. However, since some rats did not respond to IMI therapy, we hypothesize that chronic administration of this drug to rats exposed to CMS may differently affect antioxidant system in responding and non-responding animals. According to currently available literature, there are no studies providing the data which may help to relate IMI treatment resistance to oxidative system dysfunction in the distinct target tissue, such as the liver.

To check the above-postulated hypothesis, in the present study, we examined the effects of CMS and chronic IMI treatment, alone or in combination, on markers of oxidative stress, such as production of ROS, lipid peroxidation measured by malondialdehyde (MDA) level, concentrations of antioxidants, i.e., glutathione (GSH) assessed by non-protein sulfhydryl groups (NPSH) and sulfane sulfur (SS) in the rat liver. SS is a labile and highly reactive sulfur in 0 or −1 oxidation state covalently bound to another sulfur atom formed during the anaerobic cysteine metabolism. It shows regulatory and more potent antioxidant properties than GSH (Everett et al. 1994; Iciek and Włodek 2001). To further characterize severity of oxidative stress in these groups of rats, the activities of key antioxidant enzymes: catalase (CAT), glutathione peroxidase (GPx), and superoxide dismutase (SOD) were also determined in the rat liver. We hope that this set of experiments will shed a new light on a role of IMI in the treatment of drug-resistant depression.

## Materials and Methods

### Animals

Behavioral tests were carried out on male Wistar rats (Charles River, Germany), of initial body weight 220–240 g (about 7 weeks old). The animals were kept singly housed under standard laboratory conditions with free access to standard laboratory food and tap water, at room temperature of 22 °C with an artificial day–night cycle (12/12 h, light on at 8 a.m.). Each experimental group consisted of 6–8 rats.

All the procedures were carried out in accordance with the National Institutes of Health Guide for the Care and Use of Laboratory Animals and were granted an approval from the Bioethics Commission as compliant with Polish Law. All the experimental procedures were approved by the Local Bioethics Commission of the Institute of Pharmacology, Polish Academy of Sciences in Krakow.

### Drug

Imipramine hydrochloride (IMI, Sigma-Aldrich, USA) was obtained commercially, dissolved in sterile 0.9 % NaCl solution, and injected at a dose of 10 mg/kg b.w. i.p. in a volume of 1 ml/kg.

### Chronic Mild Stress (CMS) Procedure

Male wistar rats were brought into the laboratory a month prior to the start of the experiment. The animals were first trained to consume a 1 % sucrose solution. Training consisted of six 1-h baseline tests once a week in which sucrose was presented in the home cage following 14-h periods of food and water deprivation. The sucrose intake was measured by weighing the bottles before and at the end of the test and subtraction of results. After the training period, sucrose consumption was further monitored at weekly intervals throughout the experiment. On the basis of their sucrose intakes in the final baseline test, the animals were divided into two matched groups. One group of animals was subjected to the CMS procedure for a period of seven consecutive weeks. Each week the stress regime consisted of two periods of food or water deprivation, two periods of 45° cage tilt, two periods of intermittent illumination (lights on and off every 2 h), two periods of soiled cage (250 ml water in sawdust bedding), one period of paired housing, two periods of low-intensity stroboscopic illumination (150 flashes/min), and three periods of no stress. All stressors were 10–14 h in duration and were applied individually and continuously. Control animals were housed in separate rooms and had no contact with the stressed animals. They were deprived of food and water for 14 h preceding each sucrose test, but otherwise food and water were freely available in the home cage.

On the basis of their sucrose intakes following initial 2 weeks of stress, both the stressed and the control groups were each divided further into matched subgroups, and for subsequent five weeks they received once daily intraperitoneal injections of vehicle (sterile saline, 1 ml/kg b.w.) or IMI (10 mg/kg b.w.). IMI was administered at approx. 10.00 a.m. and the weekly sucrose tests were carried out 24 h following the last drug injection. Stress was continued throughout the entire period of treatment. After seven weeks of stress, sucrose intake was significantly lower in the stressed animals, but administration of IMI for 5 weeks to the animals still subjected to CMS resulted in most of animals in a significant reduction in anhedonia (CMS IMI-reactive, CMS IMI-R), as measured by sucrose intake, which remains in agreement with previously published data (Monleon et al. 1995). About 20 % of animals did not respond to the imipramine treatment by reversal of stress-induced sucrose intake deficit and we called this group IMI non-reactive (CMS IMI non-reactive, CMS IMI-NR).

### Chemicals

5,5′-Dithiobis-2-nitrobenzoic acid (DTNB), GSH reduced (GSH), 2-thiobarbituric acid (TBA), GSH reductase (GR), NADPH, epinephrine (adrenaline), 2′,7′-dichlorofluorescein diacetate (DCFH-DA), potassium cyanide (KCN), trichloroacetic acid (TCA) were provided by Sigma Chemical Co. (St. Louis, MO, USA). Formaldehyde and hydrogen peroxide (H_2_O_2_) were obtained from the Polish Chemical Reagent Company (P.O.Ch, Gliwice, Poland).

### Preparation of Tissue Homogenate

After termination of behavioral procedure (i.e., 2 days after the final sucrose intake test), rats were killed by decapitation, their livers were dissected and immediately frozen on dry ice. Then, the tissues were stored at −80 °C until further procedures were applied.

The frozen livers were weighed, and homogenates were prepared by homogenization of 1 g of the tissue in 4 ml of 0.1 M phosphate buffer, pH 7.4 using an IKA-ULTRA-TURRAX T8 homogenizer. Liver homogenates were next used for assay of the activity of antioxidant enzymes (CAT, GPx, SOD) and some markers of oxidative stress, such as the levels of ROS, MDA, non-protein sulfhydryl groups (NPSH corresponding mainly to GSH), and sulfane sulfur.

### Determination of Reactive Oxygen Species Level

DCFH-DA was deestrified in homogenates to dichlorohydrofluorescein, and was then oxidized to fluorescent dichlorofluorescein by ROS (Bondy and Guo 1994). Briefly, 10 μl of a liver homogenate were added to 980 μl of 0.1 M phosphate buffer (pH 7.4) and 10 μl of 1.25 mM DCFH-DA in ethanol. Mixtures were incubated at 37 °C for 30 min protected from light and then centrifuged at 12,000×*g* for 8 min. Fluorescence was measured with Hitachi F-2000 fluorescence spectrometer at an excitation of 488 nm and emission of 525 nm. ROS were evaluated from a standard curve with 10 μM dichlorofluorescein.

### Determination of Malondialdehyde Level

The level of MDA as a measure of lipid peroxidation was determined using the TBA spectrophotometric assay with 1,1′,3,3′-tetraethoxypropane as a standard (Ohkawa et al. 1979). TBA reacts with some products of lipid peroxidation in acidic environment at increased temperature to form a pink compound. Briefly, 250 μl of a liver homogenate were added to 250 μl of distilled water, 500 μl of 15 % TCA, and 500 μl of 0.37 % TBA. TCA and TBA solutions were prepared in 0.25 M HCl. The samples were heated in a boiling water bath for 10 min. After cooling, the samples were centrifuged at 12,000×*g* for 10 min. The absorbance of the supernatant was measured at 535 nm.

### Determination of Non-protein Sulfhydryl Group Level

The level of NPSH was estimated with DTNB according to the method described by Sedlak and Lindsay (1968). In this assay, DTNB is reduced by non-protein sulfhydryl groups present in TCA extract to yellow 2-nitro-5-mercaptobenzoic acid, absorbance of which is measured. For the estimation of NPSH, 0.05 ml of TCA extract and 0.1 ml of 6 mM DTNB were added in succession to 0.85 ml of 0.2 M phosphate buffer pH 8.2, and absorbance was measured at 412 nm. The total content of non-protein sulfhydryl groups was determined from a standard curve for 1 mM GSH.

### Determination of Sulfane Sulfur Level

The level of the compounds containing sulfane sulfur was determined by the method of Wood (1987) based on cold cyanolysis and colorimetric detection of ferric thiocyanate complex ion. To 200 μl of liver homogenate, 80 μl of 1 M NH_3_, 620 μl of distilled water, and 100 μl of 0.5 M KCN were added. The samples were incubated at room temperature for 45 min. Then 20 μl of 38 % formaldehyde and 200 μl of Goldstein’s reagent [Fe(NO_3_) + HNO_3_ + H_2_O] were added. After centrifugation at 12,000×*g* for 10 min, the absorbance at 460 nm was determined. A standard curve was prepared with 1 mM KSCN.

### Determination of Superoxide Dismutase Activity

SOD activity was measured by a colorimetric assay. Superoxide radical anion $({\text{O}}_{2}^{ \cdot } )$, the substrate for SOD, was generated indirectly in the oxidation of epinephrine at alkaline pH by the action of oxygen. The pink oxidation product of epinephrine (adrenochrome) was measured spectrophotometrically at 485 nm (Misra and Fridovich 1972). Briefly, to 875 μl of 50 mM carbonate buffer, pH 10.2, 25 μl of liver homogenate diluted 100-fold and 100 μl of 10 mM epinephrine solution were added. The absorbance at 485 nm was measured for 3 min. As a standard 25 μl of SOD solution with known activity was used.

### Determination of Glutathione Peroxidase Activity

Activity of GSH peroxidase was assayed by the method of Flohe and Gunzler (1984). This method was based on GSH oxidation by hydrogen peroxide that was catalyzed by GPx. This reaction yielded GSSG which was then reduced by GR to GSH at the expanse of NADPH oxidation. NADPH oxidation caused the decrease in absorbance at 340 nm, which could be measured spectrophotometrically. To a thermostated spectrophotometric cuvette kept at 37 °C, the following reagents were added: 600 μl of 0.1 M phosphate buffer pH 7.0 containing 0.1 mM EDTA, 100 μl of homogenate diluted 50-fold, 100 μl of GR solution of 2.4 U/ml final activity, 100 μl of 10 mM GSH solution, and 100 μl of 1.5 mM NADPH solution in 0.1 % NaHCO_3_ solution. The reaction was initiated by the addition of 100 μl of 1.5 mM H_2_O_2_ heated to 37 °C. Then the decline in absorbance was measured at 340 nm for 2 min. The difference between absorbance decrement (Δ*A*_340_/min) in the homogenate-containing sample and control sample (without homogenate) was calculated. The difference between the absorbance change rates is a measure of GPx activity in the sample.

### Determination of Catalase Activity

Catalase activity was determined by the Aebi (1984) method. Catalase degrades H_2_O_2_, which can be measured directly by the decrease in the absorbance at 240 nm. In this method, 50 μl of liver homogenate diluted 50-fold was added to 650 μl of 50 mM phosphate buffer, pH 7.0. The reaction was initiated by addition of 300 μl of 54 mM H_2_O_2_ and the decrease in absorbance was measured for 1 min at 25 °C. A unit of catalase activity was defined as such amount of the enzyme which decomposed 1 µmol H_2_O_2_ per minute.

### Determination of Protein Content

Protein content was assayed by Lowry’s et al. method (1951), which is based on the reaction of peptide bonds and aromatic amino acid residues of proteins with Folin–Ciocalteu reagent (a mixture of phosphotungstic acid and phosphomolybdic acid) in alkaline environment in the presence of cupric ions. Copper (II) ions, bound to protein tyrosine and tryptophan residues, reduce the above acids to oxides. Absorbance was measured at 500 nm. A 1 % solution of bovine albumin was used to prepare a standard curve.

### Statistical Analysis

A statistical analysis of experimental data was performed using a one-way or two-way ANOVA followed (if significant) by Duncan test for post hoc comparisons. For each set of data presented in Figs. 3a, b, 4a, b, and 5a–c, two-way ANOVA was carried out two times. First time for the following 4 groups of rats: control, CMS-subjected, chronically treated with IMI, CMS-subjected responding to IMI (CMS IMI-R), and second time for the following combination of four groups of rats: control, CMS-subjected, chronically treated with IMI, CMS-subjected non-responding to IMI (CMS IMI-NR). The results are presented as the mean ± SEM, the differences were considered statistically significant when *p* < 0.05.

## Results

### The Effects of Chronic Mild Stress and Imipramine Treatment on Sucrose Consumption

**Fig. 1:**
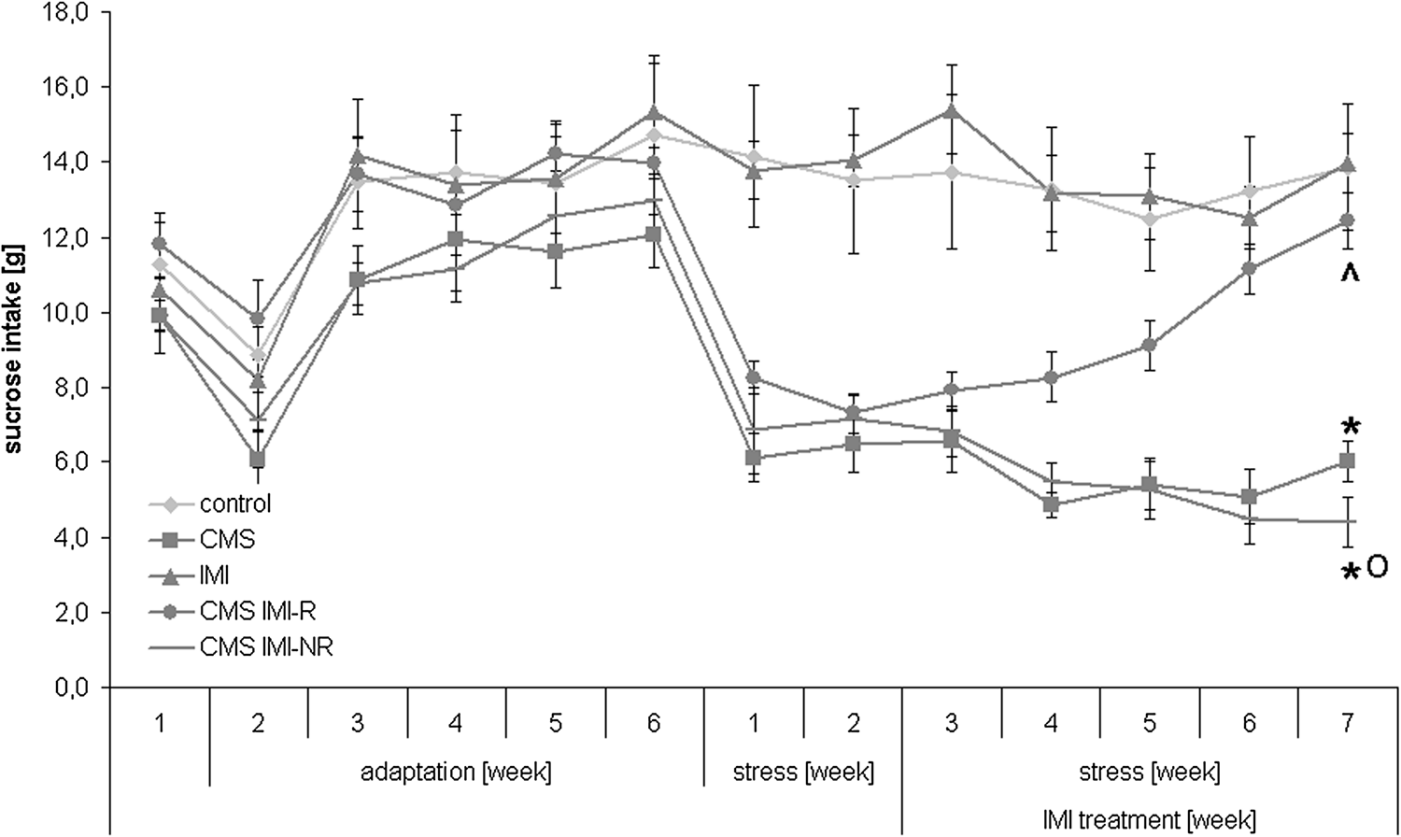
Sucrose intake during six weeks of adaptation and seven weeks of chronic mild stress (CMS) procedure involving five weeks of imipramine (IMI) administration. Data represent the means ± SEM; *n* = 7, *p < 0.05 versus control, ^*p* < 0.05 versus CMS, ^o^ *p* < 0.05 versus CMS IMI-R

For illustrative purposes, Fig. 1 shows the levels of sucrose intake during entire period of behavioral procedure, involving training for sucrose consumption terminated by baseline test after six weeks of adaptation, two weeks of stress procedure without any drug administration, and further five weeks of stress regimen accompanied by chronic IMI treatment.

**Fig. 2:**
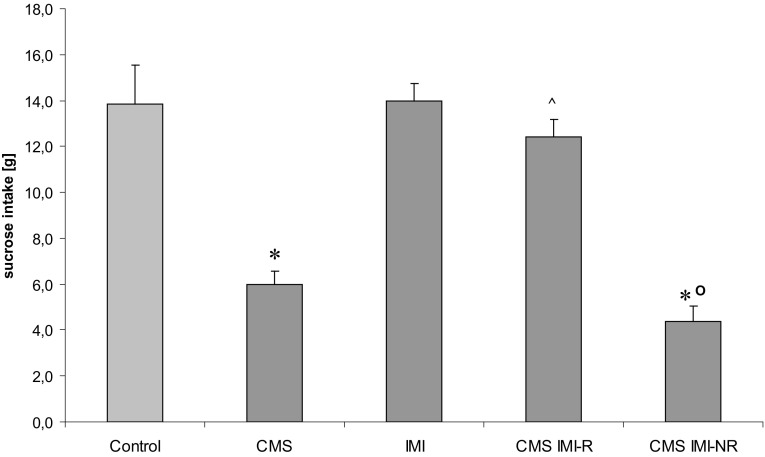
The effect of 7-week exposure to CMS and 5-week treatment with IMI on the consumption of a 1 % sucrose solution. After seven weeks of stress and five weeks of IMI treatment, the sucrose intake was statistically significantly different between the CMS IMI-R group and CMS IMI-NR animals. Data represent the means ± SEM; *n* = 7, *p < 0.05 versus control; ^*p* < 0.05 versus CMS; ^o^ *p* < 0.05 versus CMS IMI-R

In rats subjected to CMS for 7 weeks, sucrose intake was significantly lower than in non-stressed animals, amounting to 6.00 ± 0.50 versus 13.90 ± 1.7, respectively, [*F*(1,18) = 19,885, *p* < 0.005]. Administration of IMI for 5 weeks to the animals continuously exposed to CMS resulted in significant reduction in anhedonia, as measured by sucrose intake, in CMS IMI-R animals and lack of such reduction in CMS IMI-NR rats (Fig. 2).

### The Effects of Chronic Mild Stress and Imipramine Treatment on Parameters of Oxidative Stress (ROS and MDA)

**Fig. 3:**
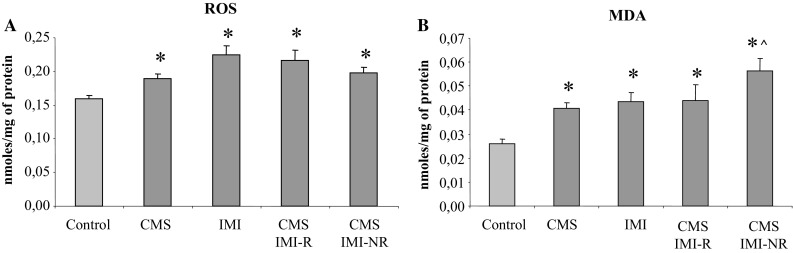
The effect of 7-week exposure to CMS and 5-week IMI treatment on reactive oxygen species (ROS) (**a**) and malondialdehyde (MDA) level (**b**) in the liver. Data represent the means ± SEM; *n* = 7, *p < 0.05 versus control; ^*p* < 0.05 versus CMS

**Fig. 4:**
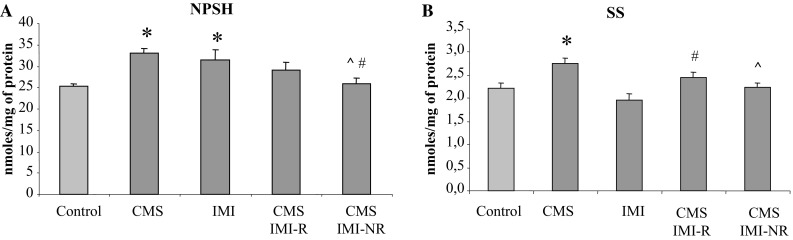
The effect of 7-week exposure to CMS and 5-week IMI treatment on non-protein sulfhydryl groups (NPSH) level (**a**) and sulfane sulfur (SS) level (**b**) in the liver. Data represent the means ± SEM; *n* = 7, *p < 0.05 versus control; ^*p* < 0.05 versus CMS, ^#^ *p* < 0.05 versus IMI

**Fig. 5:**
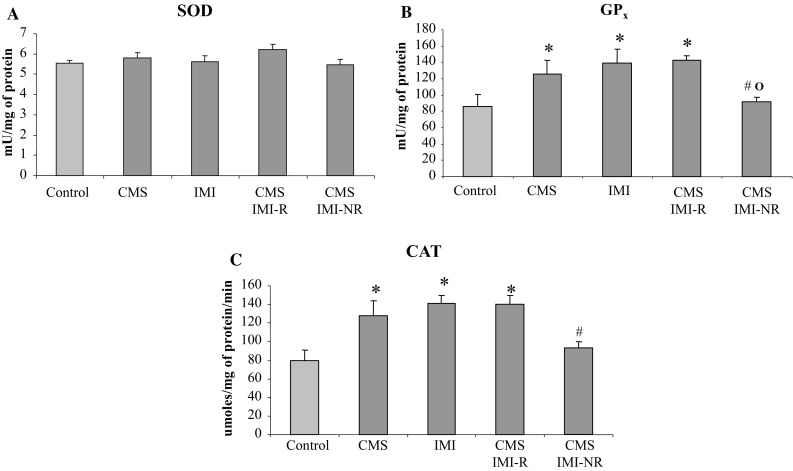
The effect of 7-week exposure to CMS and 5-week IMI treatment on superoxide dismutase (SOD) (**a**), glutathione peroxidase (GPx) (**b**), and catalase (CAT) (**c**) activity in the liver. Data represent the means ± SEM; *n* = 7, *p < 0.05 versus control, ^#^ *p* < 0.05 versus IMI, ^o^ *p* < 0.05 versus CMS IMI-R

In general, for each tested parameter presented in Figs. 3, 4, and 5, a two-way ANOVA was performed twice; in the first variant (V1) four groups were analyzed: control, CMS, IMI, and CMS IMI-R while in the second variant (V2) the groups: control, CMS, IMI, and CMS IMI-NR were compared.

Regarding ROS level, this analysis calculated for V1 revealed a significant treatment effect of IMI [*F*(1,34) = 22.875, *p* < 0.001], a lack effect of CMS application (*F* = 1.376, NS) and no interaction between IMI and CMS (*F* = 3.932, *p* = 0.055; Fig. 3a). The same analysis for V2 showed no effect of CMS (*F* = 0.029, NS), significant treatment effect of IMI [*F*(1,34) = 17.122, *p* < 0.001], and interaction of CMS × IMI (*F* = 10.862, *p* < 0.01; Fig. 3a).

A two-way ANOVA performed for MDA levels in the first variant showed a significant effect of IMI (*F* = 7.416, *p* < 0.01), lack of effect of CMS (*F* = 3.761, *p* = 0.06), and no interaction between CMS × IMI (*F* = 3.380, NS). The same analysis for variant 2 revealed both treatment effect of IMI (*F* = 22.858, *p* < 0.0001) and CMS (*F* = 15.272, *p* < 0.001) but no interaction of IMI × CMS (*F* = 0.089, NS).

Altogether, the present studies indicated that application of CMS procedure and repeated IMI administration significantly increased ROS concentrations (by 19.5 and 41.5 %, respectively; Fig. 3a) and lipid peroxidation assessed in liver homogenates by malondialdehyde levels (by 56 and 67 %, respectively; Fig. 3b) compared to the control group.

In stressed, IMI non-reactive rats, an excessive accumulation of the aldehyde products of lipid peroxidation (MDA) was found compared to their contents in control and CMS-subjected rats (by ca. 115.5 and 38 %, respectively; Fig. 3b). However, in CMS IMI-R rats, an increase in MDA level was less pronounced (by 68 % of control and 8 % of CMS-subjected groups, Fig. 3b).

### The Effects of Chronic Mild Stress and Imipramine Treatment on the Levels of Antioxidants

As to NPSH level, a two-way ANOVA performed for variant 1 demonstrated no treatment effect of IMI (*F* = 2.815, NS) and CMS application (*F* = 0.532, NS) but a significant interaction of CMS × IMI (*F* = 10,711, *p* < 0.002). Similarly, for variant 2 this analysis revealed lack of the effect of IMI (*F* = 0.573, NS) and CMS (*F* = 0.080, NS) but a marked interaction between CMS × IMI (*F* = 21,876, *p* < 0.001).

The levels of non-protein thiols (NPSH), mainly represented by GSH were significantly increased in groups subjected to CMS (by 31 %) or chronic IMI treatment (by 25.3 %) compared to that in control group. In the CMS IMI-R, only a small non-significant increase in NPSH level (15 % of control) was observed while in CMS IMI-NR animals it was significantly decreased in comparison to CMS and IMI-treated groups (Fig. 4a).

A two-way ANOVA performed for SS levels in the first and second variants revealed significant effects of CMS application (*F* = 20.917, *p* < 0.001 and *F* = 13.797, *p* < 0.001, respectively) and chronic IMI treatment (*F* = 5.7, *p* < 0.05 and *F* = 11.854, *p* < 0.01, respectively) and a lack of interactions CMS × IMI (*F* = 0.063, NS and *F* = 1.552, NS, respectively).

Application of CMS induced a marked increase in SS content (by ca 25 % of control) while chronic IMI treatment decreased it in a non-statistically significant manner (by 11 % of control). In the CMS IMI-R, a significant increase in SS content (by 25 % of IMI-treated group) was observed while in CMS IMI-NR rats, its level was slightly but non-significantly elevated (by 14 % of IMI-treated group) and significantly decreased in comparison to CMS-subjected rats (Fig. 4b).

### The Effects of Chronic Mild Stress and Imipramine Treatment on Activity of Antioxidant Enzymes

There was no significant effect of chronic IMI administration and/or CMS procedure on SOD activity in the rat liver (Fig. 5a).

A two-way ANOVA performed for GPx activity in the rat liver showed in the first variant a significant effect of chronic IMI treatment (*F* = 6.111, *p* < 0.02), a lack of effect of CMS (*F* = 2.423, NS), and no interaction between CMS × IMI (*F* = 1.709, NS). The same analysis for variant 2 revealed only a significant interaction of CMS × IMI (*F* = 8.544, *p* < 0.01) but a lack of effects of CMS procedure (*F* = 0.053, NS) and chronic IMI treatment (*F* = 0.398, NS).

Both CMS and chronic IMI treatment increased GPx activity (by 47 and 62 % of control, respectively). In the CMS IMI-R rats, the activity of this enzyme was still significantly enhanced (by 66 % of control). On the other hand, in the CMS IMI-NR rats, a significant decrease in GPx activity was observed compared to rats subjected to chronic IMI treatment or to rats subjected to CMS reactive to IMI (by 27 and 34 %, respectively) (Fig. 5b).

A two-way ANOVA performed for CAT activity in the rat liver showed in the first variant significant effects of CMS (*F* = 4.709, *p* < 0.05) and chronic IMI treatment (*F* = 11.073, *p* < 0.06) and an interaction of CMS × IMI (*F* = 5.075, *p* < 0.05). The same analysis for variant 2 revealed only a significant interaction of CMS × IMI (*F* = 16.625, *p* < 0.001) but lack of the effects of CMS procedure (*F* = 0.467, NS) and chronic IMI treatment (*F* = 3.733, *p* = 0.06).

Similarly as in the case of GPx, CMS and chronic IMI treatment increased CAT activity (by 61 and 77 % of control, respectively). In the CMS IMI-R rats, the activity of this enzyme was still significantly enhanced (by 76 % of control) while in CMS IMI-NR rats it was markedly decreased compared to group of non-stressed rats receiving chronic IMI (by 25 %) (Fig. 5c).

## Discussion

The present studies confirmed that CMS procedure models one of the main symptom of depression, namely anhedonia, and administration of IMI for 5 weeks to the animals still subjected to CMS resulted in a significant reduction in anhedonia in a majority of animals (Papp and Moryl 1994; Kubera et al. 1996), although, as it was described by some of us earlier, about 20 % of animals did not respond to the IMI treatment by reversal of CMS-induced decrease in sucrose intake (Faron-Górecka et al. 2014). Clinically, the persistence of anhedonia is one of the most treatment-resistant symptoms of major depression. In humans, only 47–52 % of patients with major depression positively responded to an initial antidepressant medication (Rush et al. 2011).

In depressed patients, total oxidative status, oxidative stress index, and markers of lipid peroxidation are increased in plasma, serum, or urine (Smaga et al. 2015). MDA levels were particularly high in patients diagnosed with recurrent depressive disorder compared to patients suffering from the first episode (Stefanescu and Ciobica 2012; Rybka et al. 2013; Lopresti et al. 2014). Post-mortem studies revealed increased lipid peroxidation in the anterior cingulated cortex of depressed patients (Wang et al. 2009). Decreased antioxidant status in depression, characterized by a significantly reduced level of non-enzymatic antioxidant molecules, such as tryptophan, tyrosine, vitamin E, ascorbic acid, alfa-tocopherol, zinc, and GSH (Kodydková et al. 2009; Maes et al. 2011a), is usually connected with a fall in GPx, CAT, and SOD activity in blood although in some other studies an increase or no differences in GPx and SOD activity were described (Bilici et al. 2001; Galecki et al. 2009; Rybka et al. 2013; Smaga et al. 2015).

Clinical studies of the effect of antidepressant drugs on oxidative biomarkers also have not brought unequivocal results. The studies of oxidative biomarkers in patients suffering from drug-resistant depression are limited to determination of the level of the antioxidant coenzyme Q10 (Maes et al. 2009). For obvious reasons, there are also no data on changes in antioxidant system function in the liver of depressed patients treated with antidepressant drugs. Therefore, we decided that it would be significant to carry out such studies in an experimental model of depression including the animals non-responding to the antidepressant action of IMI.

The above-mentioned clinical data are in line with results of the present study, which showed that rats subjected to CMS, a well-described animal model of depression, showed higher production of ROS and enhanced lipid peroxidation in the liver. These changes could lead to an adaptive response reflected by mobilization of antioxidant defense system i.e., by elevation of NPSH and SS levels as well as increase in GSH peroxidase and catalase activity in this tissue. The studies conducted so far most often examined lipid peroxidation in different animal models of depression and were focused on brain structures. All those studies reported an increase in this parameter (Smaga et al. 2015). The CMS-induced increase in ROS and lipid peroxide level in the liver might be correlated with similar oxidative changes in brain structures reported by other authors who used the same animal model of depression (Lucca et al. 2009a, b). Furthermore, Zafir and Banu (2007) using the restraint stress model found an enhanced lipid peroxidation level not only in the brain but also in the liver of the stressed animals.

Surprisingly, in the present study, chronic IMI treatment resulted in similar changes in both oxidative stress parameters and antioxidant mechanism as those evoked by the CMS, but no additive effect was observed when these two procedures were combined in IMI responding rats. The lack of additive effects of CMS and chronic IMI treatment on oxidative stress and antioxidant defense system in the liver suggests involvement of a common mechanism although this mechanism is unknown. The increase in MDA level was almost identical in CMS group, IMI group, and in CMS IMI-R group, whereas in CMS IMI-NR group, the level of MDA was significantly higher than in rats subjected to CMS. The latter effect was probably due to summing of the pro-oxidative effects of CMS and IMI resulting from the stress-induced activation of hypothalamic—pituitary—adrenal (HPA) axis and sympathetic—adrenal—medullary (SAM) axis and IMI-induced increase in peripheral level of serotonin and noradrenaline. The abnormal release of corticosteroids induces production of ROS under stress conditions (Madrigal et al. 2006; Lin et al. 2004), whereas monoaminooxidase (MAO) present in hepatocytes by deamination of monoamines also generates high number of free radicals (Wąsik et al. 2013; Antkiewicz-Michaluk et al. 2014). Removal and metabolic degradation of catecholamines by the liver plays a major role in the elimination of these amines from the circulation. Before being metabolized by enzymes inside hepatocytes, catecholamines must enter the cells, and ex vivo studies showed that IMI and its metabolite desipramine did not inhibit monoamine uptake by hepatocytes (Martel et al. 1998). In CMS IMI-R rats, the above-mentioned additive effect is not observed probably due to the inhibitory action of IMI on the HPA axis (Dhingra and Bansal, 2015) which eliminates stimulatory effect of glucocorticoids on the production of ROS.

In opposite to our results for the liver, Melzacka et al. (1995) and Reus et al. (2010) observed reduction in lipid peroxidation in the studied brain structures after acute and chronic IMI administration. Moreover, Zafir et al. (2009) showed that IMI normalized the accumulation of lipid peroxidation product (measured by malondialdehyde level) increased in the brain in response to exposure of animals to stress. On the other hand, Omar et al. (2011) and Abdel-Salam et al. (2013) observed that IMI pretreatment significantly increased brain lipid peroxidation induced by thioacetamide or lipopolysaccharide (LPS) administration and this effect was IMI dose-dependent.

In clinical practice, a prolonged treatment with antidepressants reduced MDA level, however, this observation was performed for selective serotonin reuptake inhibitors (SSRI) (Bilici et al. 2001; Khanzode et al. 2003; Galecki et al. 2009) not for IMI; moreover, no such change was observed after shorter treatment period with venlafaxine, reboxetine, or sertraline (Sarandol et al. 2007).

The sum of low molecular weight thiols (in reduced form), such as GSH, homocysteine, cysteine, and cysteinylglycine is referred to as non-protein sulfhydryl groups (Bilska-Wilkosz et al. 2013). Sulfane sulfur is defined as a labile, exceptionally reactive sulfur atom which can easily leave structure of sulfane sulfur-containing compounds and has properties of a ROS scavenger (Bilska-Wilkosz et al. 2013). The present studies showed that chronic stress affects sulfur metabolism, significantly increasing hepatic production of SS and NPSH, whereas chronic IMI administration to CMS IMI-NR, but not to CMS IMI-R rats, decreased the level of these two protective compounds in comparison to chronically stressed rats.

GSH peroxidase catalyzes the reduction of hydroxyperoxides by GSH and its main function is to protect the cell against the damaging effect of endogenously formed hydroxyperoxides. Enzymatic activity of GPx was induced by IMI, regardless of whether it was applied to unstressed or stressed animals, although CMS IMI-NR animals did not show an increase in GPx activity. Our observations are partially in agreement with those of Djordjevic et al. (2011) who showed that chronic fluoxetine administration, like chronic IMI administration in our study, increased GRx activity in the liver in chronically stressed fluoxetine-responding and unstressed animals. The increase in GPx activity emerges as a compensatory response to the enhanced lipid peroxidation since GPx scavenges primarily lipid peroxides (Djordjevic et al. 2011). The increased lipid peroxidation in CMS IMI-NR rats above the level observed in CMS IMI-R rats may be connected with the lack of increase in GPx activity in these animals.

In clinical studies in depressed patients after treatment with antidepressants, a decrease, an increase, or no differences in GPx level were observed (Bilici et al. 2001; Galecki et al. 2009; Rybka et al. 2013). The lack of the CAT activity increase in CNS IMI-NR animals, in contrast to all remaining groups, observed in our studies, like the above-described lack of GPx activity elevation, can be one of causes to drug-resistance, although this suggestion requires further studies.

In our former studies on immunity of rats resistant to antidepressant effect of IMI, we found a significant increase in IFN-γ and IL-6 synthesis by spleen cells of these animals as well as increase in inflammatory cytokine expression, including IL-6, in the hippocampus and prefrontal cortex in comparison to CMS IMI-R rats. On the other hand, it is established that pro-inflammatory cytokines, including IL-6, aggravate the oxidative damage of the vital organs, particularly the liver (Sundaram et al. 2014; Zhou et al. 2015). Kayanoki et al. (1994) showed that IL-6 suppressed catalase gene expression in cultured rat hepatocytes. It has also been shown that inhibition of CAT activity by IL-6 is nitric oxide-dependent by interaction of nitric oxide with the heme group of CAT and it was proposed that this inhibition might increase susceptibility to cytokine- or nitric oxide-induced cell killing (Sigfrid et al. 2003).

In the light of the above observations, we believe that the decrease in CAT activity in CMS IMI-NR rats compared with CMS IMI-R animals, described in the present paper, may be connected with the observed increase in pro-inflammatory cytokine level in these animals.

In the present study, we did not observe changes in SOD activity in any of the studied groups. On the other hand, Djordjevic et al. (2011) observed down-regulation of SOD activity after fluoxetine treatment in the brain of both stressed and non-stressed animals, whereas other authors reported that treatment with fluoxetine or IMI could prevent stress-induced reduction of SOD activity in the brain and liver (Zafir and Banu 2007; Zafir et al. 2009). SOD plays a predominant role in scavenging superoxide anion transforming it into hydrogen peroxide. Even though the activity of SOD is not changed after chronic IMI and/or chronic stress application, our results showed an increase in activity of the hydrogen peroxide-metabolizing enzymes, like catalase and GSH peroxidase.

In clinical reports, the effect of antidepressant drug treatment on SOD activity is not clear. SOD activity was lowered (Djordjevic et al. 2011), not changed (Kotan et al. 2011), or increased (Herken et al. 2007) in patients treated with antidepressant drugs.

In summary, taking into account the results of behavioral studies described in the present paper, we suggest that oxidative stress and antioxidant system in the liver, in contrast to the brain (Liu et al. 1996; Lucca et al. 2009a, b; Możdżeń et al. 2014), are not directly connected with anhedonia and antidepressant effect of IMI. In opposite to our expectation, the CMS-induced hepatic oxidative stress was not prevented by the IMI administration. CMS induced similar destructive changes in oxidative system in the liver as chronic administration of IMI to control or stressed animals. This investigation revealed that the restorative actions of IMI as evidenced by the reduction of anhedonia in CMS IMI-R rats were accompanied by the increase in ROS level and lipid peroxidation, and similar increase in both these destructive parameters was observed also as a result of CMS or chronic IMI administration to control animals. Moreover, in chronically stressed and IMI-treated animals which did not respond to antidepressant activity of IMI, MDA level was increased in comparison to CMS rats, activity of antioxidant enzymes (GPx and CAT) was decreased in comparison to animals chronically treated with IMI, whereas levels of two protective compounds SS and NPSH were decreased in comparison to chronically stressed rats. At present, there is no direct answer to the question whether low reactivity of antioxidant system in the liver of the non-responder group could participate, at least partly, in the mechanism of resistance to IMI.

In the present study, we evidenced a pro-oxidant action of IMI on the liver, particularly evident in IMI non-responding rats. This poses the question about a disadvantageous action of this drug not only in depression but also in comorbid diseases. The clinical significance of this observation remains to be established, especially because IMI is used for therapy of enuresis in children, and in view of the fact that antidepressant drugs are used for the treatment of depression in patients, with comorbid cancer or neurological and/or cardiologic diseases underlain, like depression, by oxidative stress, and in patients with hepatitis C viral infection, particularly those treated with interferon alpha/ribavirin combination therapy (Abdel Salam et al. 2013).

The obtained results suggest that IMI therapy in patients resistant to this drug should be discontinued immediately because it is dangerous to the correct functioning of the liver.
